# Targeted Therapies for Brain Metastases from Breast Cancer

**DOI:** 10.3390/ijms17091543

**Published:** 2016-09-13

**Authors:** Vyshak Alva Venur, José Pablo Leone

**Affiliations:** Division of Hematology, Oncology, Blood and Bone Marrow Transplantation, Department of Internal Medicine, University of Iowa Hospitals and Clinics, Iowa City, IA 52242, USA; vyshak-alvavenur@uiowa.edu

**Keywords:** brain metastases, breast cancer, HER2, VEGF, PI3K, mTOR, EGFR, CDK-4/6

## Abstract

The discovery of various driver pathways and targeted small molecule agents/antibodies have revolutionized the management of metastatic breast cancer. Currently, the major targets of clinical utility in breast cancer include the human epidermal growth factor receptor 2 (HER2) and epidermal growth factor receptor (EGFR), vascular endothelial growth factor (VEGF) receptor, mechanistic target of rapamycin (mTOR) pathway, and the cyclin-dependent kinase 4/6 (CDK-4/6) pathway. Brain metastasis, however, remains a thorn in the flesh, leading to morbidity, neuro-cognitive decline, and interruptions in the management of systemic disease. Approximately 20%–30% of patients with metastatic breast cancer develop brain metastases. Surgery, whole brain radiation therapy, and stereotactic radiosurgery are the traditional treatment options for patients with brain metastases. The therapeutic paradigm is changing due to better understanding of the blood brain barrier and the advent of tyrosine kinase inhibitors and monoclonal antibodies. Several of these agents are in clinical practice and several others are in early stage clinical trials. In this article, we will review the common targetable pathways in the management of breast cancer patients with brain metastases, and the current state of the clinical development of drugs against these pathways.

## 1. Introduction

Brain metastases are an all too frequent occurrence in metastatic breast cancer patients, with approximately 10%–16% of patients developing symptomatic brain metastases and another 10% of patients noted to have asymptomatic brain involvement in post-mortem autopsies [1,2]. In fact, breast cancer is the second most common cause of brain metastases, only behind lung cancer. The prevalence of breast cancer patients with brain metastases (BCBM) is increasing due to considerable improvements in overall survival and better imaging studies, and partly due to poor central nervous system (CNS) penetration of the existing systemic therapeutic agents. It is evident that certain malignancies such as lung cancer, melanoma, and breast cancer have a higher propensity of brain metastases when compared to colon and prostate cancer. Moreover, even among breast cancer patients, human epidermal growth factor receptor 2 (HER2) overexpressing and triple negative breast cancer have higher incidences of brain metastases compared to luminal A and B subtype. The pathophysiology behind this neurotropism has been a focus of intense research. Upregulation of certain signaling pathways like WNT/β-catenin signaling might partially explain the phenomenon [3]. Although studies so far have failed to show that brain metastases lead to mortality, these patients often have concomitant progressive extracranial disease limiting their survival. The median overall survival for BCBM patients ranges from 2 to 25.3 months [4]. Given this broad range for survival, various prognostic factors and indices have been postulated [5]. In BCBM patients, the critical prognostic factors are age, tumor subtype (triple negative vs. HER2 vs. luminal), Karnofsky performance status (KPS), number of brain metastases, and the presence of extracranial metastases [6,7]. The breast cancer specific graded prognostic index is the most recent and frequently used prognostic tool which incorporates the above mentioned factors [8]. Surgery, whole brain radiation therapy (WBRT), and stereotactic radiosurgery (SRS) are the three major treatment options for BCBM, with systemic therapy taking a backseat. Surgery is used for solitary or up to three brain metastases. It leads to the prompt resolution of peritumoral edema and provides significant symptom relief. Clinical trials comparing surgery to WBRT have shown survival benefits and better neurological functions with surgery when compared to WBRT alone [9]. Post-operative radiation therapy (either WBRT or SRS) improves intracranial disease control and even prolongs survival in those with well controlled extracranial disease [10,11]. WBRT plays an important role in the management of multiple brain metastases. SRS is a relatively new therapeutic modality which can be used in patients with four or fewer intracranial lesions. WBRT leads to significant neuro-cognitive decline compared to SRS, however, it provides better CNS control [12]. Traditionally, chemotherapy played a limited role in the management of BCBM. The blood brain barrier (BBB) with its tight junctions limits the passage of large molecules from the blood to the brain [13]. In addition, the BBB harbors various ATP binding cassette efflux transporters including P-glycoprotein and breast cancer resistance protein (BCRP), which bind to structurally diverse drugs and render them ineffective [14]. However, recent studies have led to major advances in the understanding of the BBB. There is evidence indicating that different tumor types and subtypes lead to various degrees of disruption of the BBB; for example, studies suggest that triple negative breast cancer patients have more disruption in the BBB with brain metastases then HER2 positive patients [15]. There is also the realization that once the BBB is breached by brain metastases a different blood tumor barrier can be formed which in turn might lead to limited drug delivery [16]. Additionally, we have learnt that the brain is not an immune privileged organ; it initiates and regulates immune responses and the BBB plays an important role in this process [17]. Armed with an ever improving understanding of the dynamics of brain metastases and the BBB, several existing and novel agents have been shown to possess intracranial activity. In this article, we will review the evidence behind the use of novel targeted agents in brain metastases from breast cancer. These agents act against pivotal pathways in breast cancer, and many of them have been approved for the management of advanced breast cancer. However, most of the initial trials with these agents excluded patients with brain metastases, hindering their development in the management of BCBM.

## 2. HER2 Pathway in Breast Cancer Brain Metastases (BCBM)

The HER2 pathway is one of the most researched pathways in the management of breast cancer. The HER2 receptor is a tyrosine kinase receptor and part of the epidermal growth factor receptor family (HER/EGFR/ERBB). It is found in approximately 20% of breast cancer patients and confers an aggressive natural course [18]. Compared to other types of breast cancer, HER2-positive tumors have a higher incidence of brain metastases. Up to 50% of HER2 positive breast cancer patients develop intracranial metastases [19]. Several factors have been postulated to account for the propensity of HER2-positive breast cancer to metastasize to the CNS, including the prolonged survival of patients treated with anti-HER2 therapy, the limited intracranial activity of anti-HER2 therapy, and the inherent tropism of HER2 positive breast cancer to the brain. This neuro-tropism was illustrated in preclinical in vivo models showing increased brain parenchymal colonization of metastatic HER2 positive breast cancer cells [20]. The evaluation of resected brain metastases has also revealed that the BBB was preserved in patients with HER2-positive breast cancer, despite having brain metastases [15]. Several anti-HER2 agents have been developed for clinical use. These can be classified into monoclonal antibodies (trastuzumab, pertuzumab), small molecule tyrosine kinase inhibitors (lapatinib, neratinib), and antibody-drug conjugates (T-DM1). Table 1 provides a glimpse of the most important clinical trials in HER2 overexpressing BCBM.

Trastuzumab, like most other monoclonal antibodies, does not cross the intact BBB, as elucidated by studies comparing the plasma to cerebrospinal fluid (CSF) concentration of trastuzumab [21]. Stemmler et al. in 2007 evaluated the ratio of the plasma to CSF concentration of trastuzumab in patients with brain metastases [22]. Using an immunoenzymatic test, they measured the functionally active trastuzumab in various body fluids. The CSF to plasma levels of trastuzumab in patients with brain metastases prior to any local therapy was 1:420, however, after radiotherapy the ratio improved dramatically to 1:79. This concept of increased CNS penetration of trastuzumab after disruption of the BBB by either radiation therapy or surgery has been shown by a number of radio-labelled-trastuzumab imaging studies as well [23,24]. In clinical practice, few retrospective studies have shown that trastuzumab-based therapies improve survival in patients with HER2-positive breast cancer with brain metastases, although, this survival benefit is largely due to extracranial disease control [25,26]. Pertuzumab, another monoclonal antibody that acts against a different epitope of the HER2 receptor, has been approved for use in metastatic breast cancer patients with HER2 overexpression, in combination with trastuzumab and docetaxel [27]. In a post-hoc analysis of the registration trial of pertuzumab, the CLinical Evaluation Of Pertuzumab And TRAstuzumab (CLEOPATRA) trial, the median time to development of CNS metastases as a first site of disease progression was significantly prolonged in patients treated with pertuzumab to 15.0 months, compared to the placebo group at 11.9 months [28]. Due to the difficulties with CNS penetration of monoclonal antibodies, several studies are currently underway, evaluating the role of intrathecal trastuzumab alone and in combination with pertuzumab, to explore new routes that would increase drug availability in the CNS [29].

Trastuzumab emtansine (T-DM1) is a novel antibody-drug conjugate that has improved overall survival in the Trastuzumab Emtansine vs. Capecitabine + Lapatinib in Patients with HER2-Positive Locally Advanced or Metastatic Breast Cancer (EMILIA) trial, which included previously treated HER2-overexpressing metastatic breast cancer and compared T-DM1 versus lapatinib plus capecitabine [35]. Ninety-five of the 991 patients enrolled in the EMILIA trial had CNS metastases at enrollment. A retrospective exploratory analysis of these 95 patients showed similar progression free survival in both the groups (5.9 months vs. 5.7 months), however, those treated with T-DM1 had significant improvement in overall survival (26.8 months vs. 12.9 months) [30]. This improvement in overall survival is likely due to better systemic disease control. Several studies combining T-DM1 with other systemic therapies are accruing patients [36,37]. Of particular relevance, presented at the 2016 American Society of Clinical Oncology (ASCO) Annual Meeting, Borges et al. have shown very promising results for the combination of T-DM1 with ONT-380 which resulted in sustained CNS responses [36]. ONT-380 is a small molecule tyrosine kinase inhibitor with activity against the HER2 receptor and minor, if any, activity against the EGFR receptor.

An increasing number of small molecule inhibitors are being evaluated in various phases of pre-clinical and clinical studies. Lapatinib, a tyrosine kinase inhibitor targeting both EGFR and HER2 receptors, has the ability to cross the BBB. The central nervous concentration of lapatinib varies in different contexts; in the presence of CNS metastases, the brain to plasma concentration of radio-labelled lapatinib was reported to be about 26%, whereas in normal brain parenchyma, the concentration was low at 1.3%–2.8% [38]. However, lapatinib is a substrate of efflux transporters (e.g., P-glycoprotein (Pgp) and the BCRP) located in the BBB [39]. The intracranial response rates with single agent lapatinib have been low, as shown by two phase II studies, which included HER2 overexpressing BCBM patients who progressed on trastuzumab, and reported an intracranial response rate of 3%–6% [31,40]. The intracranial response rate improved with the addition of capecitabine to lapatinib. A single arm phase II study of lapatinib and capecitabine in the treatment of naïve HER2 overexpressing BCBM patients reported a 66% intracranial response rate and a median time to intracranial progression of 5.5 months [32]. Following these encouraging results, the combinations of lapatinib and several other cytotoxic chemotherapies, such as topotecan and cabazitaxel, are currently being tested in BCBM patients [41]. Recently, another tyrosine kinase inhibitor, neratinib, was evaluated in a phase II study, where 40 patients with HER2 overexpressing BCBM who experienced CNS progression after one or more line of CNS-directed therapy were treated with a neratinib single agent [42]. The intracranial response rate was poor at 8% with a median progression free survival of 1.9 months. Several other small molecule inhibitors are currently in early phases of clinical trials. The preliminary results of phase I studies of two such molecules, ONT-380 and tesevatinib, were presented at the Annual Meeting of the American Society of Clinical Oncology in June 2016 [36,43]. ONT-380 in combination with T-DM1 is being tested in HER2 positive breast cancer patients [36]. Among the fourteen patients with BCBM enrolled in this study, the intracranial response rate was 36%. Another phase I study is investigating the activity of tesevatinib in combination with trastuzumab in HER2 overexpressing BCBM [43]. Both of these studies bring forth an interesting concept of combining a potential CNS penetrating agent with a potent agent with known extracranial activity.

## 3. Vascular Endothelial Growth Factor (VEGF) Pathway in BCBM

The vascular endothelial growth factor (VEGF) pathway plays a vital role in the development of many malignancies. In breast cancer, there was hope that VEGF inhibitors like bevacizumab would play a crucial role in improving the outcome of patients with metastatic disease, particularly in the group with HER2 negative tumors. However, multiple phase III trials and meta-analyses have failed to show any survival benefit with the addition of bevacizumab in metastatic breast cancer [44,45]. Most of these studies did not include patients with brain metastases largely due to the fear of intra-cranial hemorrhage. However, bevacizumab has been safely used in the management of primary brain tumors like glioblastoma without significant bleeding risk. In 2013, Lin et al. presented their initial findings of a phase II trial of carboplatin plus bevacizumab in BCBM [46]. At that point, 38 patients previously treated for both intracranial (WBRT or SRS) and extracranial disease had been accrued in the trial. They reported a CNS response rate of 45% by the Response Evaluation Criteria In Solid Tumors (RECIST) criteria. In another phase II study, bevacizumab was used as a conditioning regimen followed by cisplatin and etoposide for BCBM patients who had CNS progression after WBRT [47]. An intracranial response of 77% was noted in this study. The high intracranial response rates in both of the above mentioned studies must be viewed with cautious optimism, as bevacizumab leads to changes in the peritumoral vascular system, which leads to difficulties in interpreting the responses at follow up scans. This phenomenon has been frequently encountered in primary CNS tumors. Additional endpoints such as time to CNS progression may further assist in establishing the role of VEGF inhibitors in BCBM.

## 4. PI3K/Akt/mTOR Pathway in BCBM

The phosphatidylinositol3 kinase (PI3K)/Akt/mammalian target of rapamycin (mTOR) (PAM) pathway is dysregulated in several malignancies. The *PIK3CA* mutation and the deletion of *PTEN* are two of the most common aberrations in this important pathway [48,49]. In breast cancer, 28%–47% of hormone receptor positive tumors and 23%–33% of HER2 positive tumors express mutations in *PIK3CA,* whereas the loss of PTEN is seen in 29%–44% of hormone receptor positive tumors and 22% of HER2 positive tumors [50]. In contrast, 7% of basal type (triple negative) tumors harbor the *PIK3CA* mutation, and the loss of PTEN is found in 35% of triple negative breast cancer cases [51]. Everolimus, an mTOR inhibitor, and buparlisib, a PI3K inhibitor, are currently in clinical development for the management of BCBM. With promising data from the Breast cancer trials of OraL EveROlimus-2 (BOLERO-2) trial, everolimus has been approved for use in combination with an aromatase inhibitor in post-menopausal patients with hormone receptor-positive metastatic breast cancer that have progressed on or after a non-steroidal aromatase inhibitor [52]. The Breast cancer trials of OraL EveROlimus-3 (BOLERO-3) trial showed that triple therapy with vinorelbine, trastuzumab, and everolimus was superior to vinorelbine, trastuzumab, and placebo in trastuzumab-resistant advanced HER2+ breast cancer [53]. Both BOLERO-2 and BOLERO-3 excluded BCBM patients. However, CNS activity of everolimus has been shown in patients with subependymal giant-cell astrocytomas, where a phase III trial showed a 50% reduction in the size of these tumors with everolimus [54]. Various clinical trials are now evaluating the role of everolimus and buparlisib in the management of BCBM [55,56]. A single center phase Ib clinical trial plans to treat 47 BCBM patients with a combination of lapatinib, everolimus, and capecitbaine, after progressing on trastuzumab [55], and another multicenter phase II clinical trial is accruing 35 BCBM patients to evaluate the safety, tolerability, and efficacy of a combination of trastuzumab, everolimus, and vinorelbine. These and future trials will provide a better understanding of the role of targeting the mTOR pathway in BCBM. 

## 5. Epidermal Growth Factor Receptor (EGFR) Pathway in BCBM

The epidermal growth factor receptor is closely related to the HER2 receptor; both belong to the ERBB family. The EGFR inhibitors have been successfully used in advanced adenocarcinoma of the lung. First generation EGFR inhibitors, erlotinib and gefitinib, have not been tested in BCBM. In contrast, afatinib which is a second generation EGFR inhibitor has been evaluated in this group of patients. A phase II study of BCBM patients compared single agent afatinib, with afatinib plus vinorelbine, and treatment of the investigator’s choice [33]. The primary endpoint of the study was the patient benefit at 12 weeks, defined by an absence of any disease progression, no worsening of any neurological symptom, and no increase in corticosteroid use. Overall, the study failed to show any benefit with the addition of afatinib, as there was no difference in patient benefit between the afatinib containing arms and the treatment of the investigator’s choice. Therefore, the role of afatinib in BCBM still needs further investigation. Future trials may need to evaluate the status of EGFR in addition to HER2 in patients prior to the use of afatinib, to better understand the potential role of this drug and other similar targeted agents in BCBM. 

## 6. CDK-4/6 Inhibitors in BCBM

The cyclin D-CDK4/6-INK4-Rb pathway regulates the transition from the G1 (pre-DNA synthesis) to S (synthesis) phase of cell division. An intact pRb gene undergoes hyperphosphorylation during this transition, leading to the release of various transcription factors [57]. This step is crucial in the control of cell proliferation, and dysregulation of this pathway is seen in several cancers [58]. Abemaciclib, ribociclib, and palbociclib are the three CDK 4/6 inhibitors currently available. These drugs have shown high efficacy in the management of ER positive metastatic breast cancer [59] and recently there has been an interest in exploring their potential role in patients with ER positive BCBM. A recent report of abemaciclib in BCBM patients showed good CNS penetration with comparable CSF and plasma concentrations [60]. Several trials are evaluating their role in management of brain metastases [23,61]. For example, a phase II study is evaluating the safety and activity of abemaciclib in hormone receptor positive BCBM and brain metastases from lung cancer and melanoma [61]. Also, palbociclib is being tested in patients with hormone receptor positive or triple negative BCBM, in the context of a phase II clinical trial [23]. These trials will shed light on the role of this relatively new group of drugs targeting the CDK-4/6 pathway. However, CDK4/6 inhibitors appear to require an intact pRb pathway as a mechanism of action, potentially limiting their use in a significant subset of advanced breast cancers.

## 7. Conclusions

In summary, brain metastases are a major cause of morbidity in patients with advanced breast cancer. It is expected that the overall prevalence of brain metastases is going to increase in the coming years, and therefore, there is and will be an unmet need for managing intracranial and extracranial diseases in this patient population. Several new drugs with emphasis on activity in the CNS are in different stages of clinical development. Even though the majority of the drugs in the pipeline are small molecule inhibitors, innovative drug modifications are being made in traditional chemotherapies to have better BBB penetration. For example, ANG-1005, a novel peptide-drug conjugate of paclitaxel and Angiopep-2, which crosses the BBB using the lipoprotein receptor-related protein-1 (LRP-1), is being tested in an early phase clinical trial [62]. However, one disadvantage is that most of the initial clinical trials for new drugs exclude patients with BCBM, which hampers progress. Therefore, for most symptomatic patients with BCBM, local therapies such as surgery and radiation therapy (WBRT/SRS) are the standard of care. Recently, the ASCO published clinical guidelines for the management of HER2 overexpressing BCBM and emphasized the use of HER-2 targeted therapy, especially in those with extracranial progression of disease [63]. There has also been tremendous progress in the field of cancer immunotherapy and the interaction of the brain with the immune system. Several promising immunotherapeutic drugs are in development, which will be welcome additions to the arsenal of treatments used for the management of patients with brain metastases.

## Figures and Tables

**Table 1:** Pivotal clinical trials using targeted agents in the management of brain metastases (BCBM).

Study	Targeted Therapy Used	Trial Characteristics	Number of Patients	Local Control (%)	PFS (Months)	OS (Months)
Krop et al. [30]	Trastuzumab Emtasine (T-DM1) vs. lapatinib-capecitabine (XL)	Retrospective analysis of patients with CNS metastases in EMILIA trial	T-DM1: 45	Not reported	5.9	26.8
			XL: 50	Not reported	5.7	12.9
Lin et al. [31]	Lapatinib	Phase II study of progressive brain metastases, all received prior trastuzumab	39	Not reported	PFS at 4 months: 18%	NR
Bachelot et al. (LANDSCAPE) [32]	Lapatinib + capecitabine	Phase II study for Newly diagnosed brain metastases	45	65.9%	5.5	17
Cortes et al. [33]	Afatinib	Phase II three arm study. Arm A: afatinib; Arm B: afatinib plus vinorelbine; Arm C: investigators’ choice	Arm A: 40	12/40 (30%)	-	-
			Arm B: 38	13/38 (34.2%)	-	-
			Arm C: 43	18/43 (41.9%)	-	-
Freedman et al. [34]	Neratinib	Single arm phase II study in previously treated patients	40	-	1.9	8.7

PFS—Progression-free survival; OS—Overall survival; “-“—Not available.
